# Optimizing phase to enhance optical trap stiffness

**DOI:** 10.1038/s41598-017-00762-z

**Published:** 2017-04-03

**Authors:** Michael A. Taylor

**Affiliations:** 1 School of Biomedical Sciences, The University of Queensland, St Lucia, Queensland 4072, Australia; 2 Research Institute of Molecular Pathology (IMP), 1030 Vienna, Austria

## Abstract

Phase optimization offers promising capabilities in optical tweezers, allowing huge increases in the applied forces, trap stiff-ness, or measurement sensitivity. One key obstacle to potential applications is the lack of an efficient algorithm to compute an optimized phase profile, with enhanced trapping experiments relying on slow programs that would take up to a week to converge. Here we introduce an algorithm that reduces the wait from days to minutes. We characterize the achievable in-crease in trap stiffness and its dependence on particle size, refractive index, and optical polarization. We further show that phase-only control can achieve almost all of the enhancement possible with full wavefront shaping; for instance phase control allows 62 times higher trap stiffness for 10 *μ*m silica spheres in water, while amplitude control and non-trivial polarization further increase this by 1.26 and 1.01 respectively. This algorithm will facilitate future applications in optical trapping, and more generally in wavefront optimization.

## Introduction

**Figure 1:**
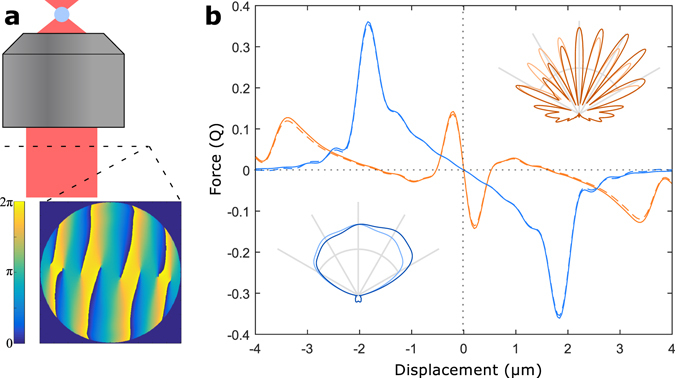
Enhanced trapping with phase-engineered light. (**a**) The system considered here has a phase profile applied at the back-focal plane of the trapping objective. This example is calculated for 3.5 *μ*m silica spheres in water with *y* polarized light. (**b**) The resulting force as a function of displacement, both for a diffraction-limited trap (blue) and with the calculated phase profile (orange). This is calculated both with the Optical Tweezers Toolbox^16^ (solid lines) and Eq. (6) (dashed lines), with the two methods showing near-perfect agreement. In this example the phase profile increases the trap stiffness by a factor of 15.5. For the remainder of the paper we use the Optical Tweezers Toolbox for all optical force and trap stiffness calculations. Insets: polar plots of the transmitted intensity after the particle, both when the particle is centered (light) and when displaced by 50 nm (dark). The diffraction-limited trap shows a small angular deflection in response to particle movement, with minimal change in the overall shape. By contrast, the particle acts to separate the phase-optimized fields into discrete interference fringes, with particle displacement causing light to be redirected between different intensity peaks, as described in ref. 6.

Optical micromanipulation relies on optical forces to precisely handle and manipulate microscopic particles. Typically this involves trapping single particles near a diffraction-limited focus^1^, though tailoring of the optical field can allow other capabilities such as a pushing optical conveyor-belt^2^, a pulling *tractor beam* ^3, 4^, or application of torque^5^. Recently it was shown that wavefront shaping also allows trap stiffness to be enhanced by over an order of magnitude when trapping large particles^6^, which could enable a range of important applications in cellular manipulation^7, 8^, fluid dynamics^9, 10^, micro-robotics^11^, and tests of fundamental physics^12, 13^. This takes advantage of non-trivial spatial structure in the scattered field, with the trapping light precisely shaped to achieve interferometric redirection of the light with small particle displacement. The resulting optical trap then exhibits characteristics analogous to a beamsplitter that mixes two incident fields, rather than the usual ray-optics picture with the particle deflecting the incident light (see Fig. 1). This characteristic behavior was referred to as ‘enhanced trapping via structured scattering’ (ENTRAPS), and was consistently observed alongside enhanced trap stiffness^6^.

The experiments in ref. 6 used a spatial light modulator to provide phase-only control of the light at the back-focal plane of the trapping objective. This is a relatively straightforward approach used widely in holographic optical tweezers, making the enhanced trapping strategy immediately achievable for many labs with existing equipment. However, there are no reported methods to efficiently optimize the optical phase for trapping applications, and ref. 6 used an extremely slow steepest descent search algorithm to calculate phase-only optima. As noted in its supplement the algorithm could take up to one week to converge when implemented on a powerful workstation computer^6^. This extremely slow performance is a major obstacle to potential applications, and also prevented careful characterization of the performance of phase-optimized traps across parameter space. Further, the algorithm could only converge on local optima that were everywhere continuous, which precludes vortices as these cannot be represented as a continuous phase surface.

Here we introduce the Sequential Phase Optimization Technique (SPOT) to efficiently optimize the phase profile, and apply this to optical trapping forces. The source code used to implement SPOT here is freely available and can be downloaded at ref. 14. This converges more than two orders of magnitude faster than the method from ref. 6, reducing the computational time from days to minutes. It allows phase singularities, which we show increases the achievable trap stiffness and allows up to 3x higher stiffness than reported in ref. 6. When trapping 10 *μ*m silica spheres in water with 1064 nm light, for instance, this allows a 62x enhancement in the trap stiffness over a Gaussian trapping field. We use SPOT to explore the dependence of the phase-optimized trap stiffness on polarization, particle size, and refractive index. We further implement the Eigenmode method which locates the globally optimal wavefront^15^, but which cannot be confined to phase-only control. By comparing the resulting optima we show that phase-only control can achieve most of the enhancement that is possible, while also being far more experimentally accessible. For sphere diameters of 1–10 *μ*m, for instance, the addition of amplitude control further increases the achievable trap stiffness by 1.35 ± 0.10, and polarization control offers only 1.02 ± 0.01 further enhancement.

### Phase optimization algorithm

Optical forces rely on scattering to redirect the momentum of the light. Optical momentum has a quadratic dependence on the electromagnetic field, while the scattering interaction is typically linear. Such interactions may be written in a general quadratic matrix form

1 $$\kappa ={{\bf{E}}}^{\ast }M{\bf{E}},$$

where *κ* is the spring constant, **E** is a column vector representing the complex electric field amplitude, *M* is a square Hermitian matrix that describes the light-matter interaction, and the superscript * denotes the Hermitian conjugate. More generally, this framework is widely applicable to linear optics and can be used to model other problems such as torque, spot size, energy flux, or peak intensity^15^.

Previously, the formalism in Eq. (1) was used in the Eigenmode method^15^. If the complex amplitude **E** is expressed as a superposition of eigenvectors of the matrix *M*, it is straightforward to show that *κ* is globally optimized when the field is populated entirely into the eigenvector that has the largest eigenvalue. This global optimum generally has non-trivial spatial structure in both the amplitude and phase, necessitating challenging wavefront shaping methods.

To analyze Eq. (1) with fixed amplitude, the *n*th element of **E** can be expressed as the product of phase ${e}^{i{\varphi }_{n}}$ and a real positive amplitude *E*_*n*_,

2 $$\kappa =\sum _{m,n}{e}^{i({\varphi }_{n}-{\varphi }_{m})}{E}_{m}{M}_{m,n}{E}_{n}\mathrm{.}$$

Because *κ* must be real, the matrix *M* is Hermitian and all imaginary components cancel. Consequently, we consider only the real part of the sum above. While it is unclear how to simultaneously optimize all phase elements, each individual element can easily be optimized. If we only control the single phase elements *ϕ*_*m*_, the trap stiffness is optimized when the product ${e}^{-i{\varphi }_{m}}{E}_{m}{\sum }_{n}{e}^{i{\varphi }_{n}}{M}_{m,n}{E}_{n}$ is real and positive. This occurs when the phase is set to

3 $${\varphi }_{m}^{{\rm{opt}}}\Rightarrow {\rm{Angle}}[{E}_{m}\sum _{n\ne m}{e}^{i{\varphi }_{n}}{M}_{m,n}{E}_{n}],$$

where we can neglect the diagonal *n* = *m* term because it does not influence *κ* (see Eq. (2)). The SPOT algorithm optimizes the full phase profile by sequentially optimizing each individual phase element according to Eq. (3). Since the ideal phase ${\varphi }_{m}^{{\rm{opt}}}$ for each mode depends on the phase of every other mode, this process must be repeated iteratively until it converges on a local optimum.

The procedure of Eq. (3) only requires a dot product followed by reading out the complex phase, which are both simple mathematical operations. This vastly reduces the computational difficulty when compared to the steepest descent approach used in ref. 6, which used optical modeling of forces at different position *x* to predict the trap stiffness *κ*, evaluated with slight changes in phase *ϕ*_*n*_ to predict $\frac{\partial \kappa }{\partial {\varphi }_{n}}$ for each mode.

### Calculation of the matrix *M*

In general the vector **E** can represent a decomposition of the electromagnetic field in any spatial mode basis. However, a phase modulation in one spatial mode basis couples to both the amplitude and phase in a different basis; as such, it is crucial to perform calculations in the spatial mode decomposition in which we control the phase. When using a spatial light modulator that is imaged to the back focal plane of the trapping objective (Fig. 1a), each pixel allows independent phase control of a plane wave at the particle. We therefore calculate *M* in the plane wave basis where each spatial mode has a unique wavevector **k**. In this basis the lateral optical momentum *p*_*x*_ is given as

4 $${p}_{x}=\hslash {{\bf{E}}}^{\ast }{K}_{x}{\bf{E}},$$

where *ħ* is Planck's reduced constant, *K*_*x*_ is the projection of the wavevector onto the *x* axis, and **E** is normalized such that E*E = *N* where *N* is the mean photon number. Within the plane-wave basis the matrix *K*_*x*_ is diagonal with the *n*th diagonal index the wavevector component along *x* of the *n*th mode (called *k*_*n*_).

When the field interacts with a particle, an optical force is applied which opposes the change in optical momentum. The interaction with the particle can be represented with a transmission matrix *T*, with the transmitted field given as **E**_*T*_ = *T* **E**. Using this in Eq. (4), the optical momentum after interaction with the particle is given as

5 $${p}_{x}=\hslash {{\bf{E}}}^{\ast }{T}^{\ast }{K}_{x}T{\bf{E}}={{\bf{E}}}^{\ast }{A}_{x}{\bf{E}},$$

where we have simplified using *A*_*x*_ = *ħT* ^*^ *K*_*x*_ *T*. For the usual case that the incident field is symmetric with no net lateral momentum, the output momentum exactly opposes the optical force *F*_*x*_ = −*p*_*x*_. To understand this equation, it is useful to note that the matrix elements of *A*_*x*_ represent the output momentum resulting from the combination of modes on the particle. Specifically, diagonal terms *A*_*m*,*m*_ denote the output momentum of the mode *m* after interaction with the particle, while off-diagonal terms *A*_*m*,*n*_ denote the change in momentum due to interference of the originally orthogonal modes *m* and *n*. For a given field, Eq. (5) can be used to immediately evaluate the force. Further, Eq. (5) has the same structure as Eq. (1) and can also be used with the SPOT algorithm above to optimize the force magnitude. It also allows evaluation of the change in force with particle displacement, since displacing the particle by **x** causes the light to arrive at the particle with phase shifted by **x** · **k**. For a lateral shift *x*, this phase shift results in an optical force of

6 $${F}_{x}(x)=-\sum _{m,n}{e}^{i({\varphi }_{n}-{\varphi }_{m})+i({k}_{n}-{k}_{m})x}{E}_{m}{A}_{m,n}{E}_{n}\mathrm{.}$$

This expression can be used to efficiently calculate force-displacement curves, with examples shown in Fig. 1. The corresponding trap stiffness is given by

7 $$\kappa ={-\frac{d{F}_{x}}{dx}|}_{x=0}=\sum _{m,n}i({k}_{n}-{k}_{m}){e}^{i({\varphi }_{n}-{\varphi }_{m})}{E}_{m}{A}_{m,n}{E}_{n}\mathrm{.}$$

Comparing this to Eq. (1), we see that the matrix *M* is given by *M*_*m*,*n*_ = *i*(*k*_*n*_ − *k*_*m*_)*A*_*m*,*n*_. We used this to implement the SPOT algorithm described above, making use of code from the Optical Tweezers Toolbox^16^ and the Quadrant Detection package^17^. The code is freely available at ref. 14. Additional details of its implementation are given in supplementary sections S1.1–S1.3, including the organization of the code (S1.1), the numerical parameters used in calculations (S1.2), and the running time of the code (S1.3). An example optimization is shown in Fig. 1, where the phase profile in Fig. 1a increases trap stiffness by a factor of 15.5 for a 3.5 *μ*m diameter silica sphere in water (Fig. 1b). The resulting trap shows the ENTRAPS characteristics that were earlier reported in ref. 6. Note that these equations do not require the net force *F*_*x*_ to be zero at *x* = 0 where the force gradient $\frac{d{F}_{x}}{dx}$ is maximized. As seen for the Gaussian trap in Fig. 1b, steep force gradients can be generated far from the trapping sites. To ensure that *x* = 0 corresponds to the stable trapping site we constrain the field to 180° rotational symmetry so that the incident field has zero net lateral momentum. This is achieved by applying the phase calculated with Eq. (3) to both the modes *m* and its image upon a 180° rotation. Stable trapping at *x* = 0 can also be ensured by enforcing *x* − *y* mirror symmetry, but we found that this results in poorer performance.

In the following sections we explore the characteristics of the phase-optimized trap, and compare this to the Eigenmode solution based on full wavefront control. Before this, we briefly note some insight that can be gained from Eq. (7). This expression can be interpreted within the picture that the particle acts as a beamsplitter to combine the modes *m* and *n*. The particle displacement provides a relative phase shift that modulates the transmitted intensity via interference. The phase shift depends on the difference in the *x* component of their wavevectors, so modes which share a similar wavevector along *x* do not contribute to the *x* axis trap.

Further, Eq. (7) places a stringent upper bound on the possible trap stiffness. The matrix *A*_*x*_ denotes the momentum per photon of the output field, so the maximum magnitude of any of its elements is *ħk*, with *k* the wavevector magnitude. Given this, the highest possible value for *κ* is

8 $${\kappa }_{{\rm{\max }}}=2\hslash {k}^{2}N=\frac{4\pi {n}_{m}^{2}P}{c\lambda },$$

where we have reparameterized using *k* = 2*πn*_*m*_/*λ* and *N* = *Pλ*/(2*πħc*), with *P* the mean power, *n*_*m*_ the medium refractive index, *λ* the vacuum wavelength and *c* the speed of light in vacuum. This upper limit lies far above any experiment to date; with 1064 nm light in water, *κ*_max_ = 70 mN/m/W. Even the experiments using state-of-the-art engineered particles with anti-reflection coatings have only achieved 3.8 mN/m/W, approximately 5% of the upper limit^18^. Here, however, we find that with wavefront shaping a homogeneous sphere can reach 26% of this upper limit. While further improvements are likely possible, for instance by engineering both the particle and the wavefront, Eq. (8) places a fundamental bound on the possible increase in trap stiffness.

The final expression in Eq. (8) is identical to that presented in the supplementary information of ref. 6, though there it was derived from the known quantum limit to displacement precision. However, engineered quantum states of light can overcome the quantum limit to particle tracking precision^19, 20^, which left open the possibility that these could also violate the limit of Eq. (8). Here the limit is derived from considerations of light propagation which provides a more robust and informative framework. This also suggests that quantum optics cannot be used to overcome the limit to trap stiffness, since quantum states of light generally propagate and diffract as expected via classical physics, with non-classical effects only observable in higher order photon statistics such as photon variance or multi-photon coincidence detection^21^. However, the limit is only applicable with propagating fields, so it may be possible to surpass it with near-field traps or optical cavities.

### Characteristics of a phase-optimized trap

**Figure 2:**
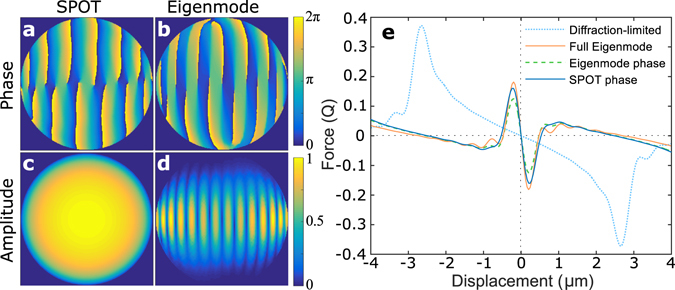
Comparison of the SPOT wavefront and the optimal wavefront calculated with the Eigenmode method, for 5 *μ*m diameter silica (n = 1.46) microspheres in water trapped with circularly polarize light. (**a**,**b**) The phase profiles of both methods include vortices; and as the Eigenmode solution is globally optimal, this must allow higher stiffness than a fully continuous phase profile. SPOT requires only a standard Gaussian amplitude (**c**), while the Eigenmode solution requires detailed amplitude structure (**d**). (**e**) The resulting trapping forces as a function of displacement. Sharp and narrow trapping features replace the wide profile of the diffraction-limited Gaussian. This increases trap stiffness without increasing the maximum achievable force. As expected the Eigenmode solution with full wavefront control provides highest stiffness, though phase-only control allows close to the same performance. Application of the Eigenmode phase (**b**) with Gaussian amplitude (**c**) provides the closest approximation to the fully optimal wavefront possible with phase-only control; however, it is clearly out-performed by our SPOT optimum.

The SPOT algorithm described above provides a powerful and efficient method to characterize phase-optimized trapping. This was implemented together with the Eigenmode method, and the performance of phase-only optimization compared to full wavefront control. In all calculations we assume the particle is in water (refractive index 1.33) and use a trapping objective with numerical aperture (NA) 1.25, vacuum wavelength of 1064 nm, and for most calculations we use silica (refractive index 1.46) microspheres. An example is shown in Fig. 2 for 5 *μ*m diameter silica spheres, which shows that the phase profile from SPOT (Fig. 2a) differs from the phase of the Eigenmode solution (Fig. 2b). While the Eigenmode phase is fully optimal when applied with the Eigenmode amplitude, it can also approximate the optimal phase profile when applied with Gaussian amplitude. This is often assumed to be the optimal phase profile, and is applied in applications such as imaging through a scattering medium^22^. However, SPOT phase-only optimization achieves 22% higher trap stiffness than application of the Eigenmode phase with Gaussian amplitude. In fact, we find that SPOT achieved higher trap stiffness than the Eigenmode phase in every set of parameters that we have calculated.

**Figure 3:**
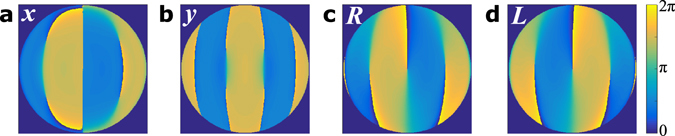
The phase of the optimal Eigenmode solutions for different polarizations. (**a**–**d**) are calculated for 2 *μ*m spheres with *x*, *y*, right circular (*R*) and left circular (*L*) respectively, with the trap optimized along the *x* axis. The optimal profiles clearly differ for the different polarizations, which could not be predicted using paraxial optics. Interestingly, the vortices in the right and left circular polarization profiles are all aligned together such that the profiles carry 3*ħ* net orbital angular momentum. These profiles are mirror-images, with the orbital angular momentum in both cases opposing the spin angular momentum carried by the polarization.

A further interesting note is that both methods show vortices in the optimized phase profiles (Fig. 2a,b). A vortex is a phase singularity around which the phase circulates by 2*π*. These vortices introduce orbital angular momentum that can be positive or negative depending on the orientation of the circulating phase. In this case all the vortices share the same orientation, such that the resulting field has a non-zero net orbital angular momentum. Since the Eigenmode method locates the global optimum, vortices must allow higher trap stiffness than a continuous phase profile. Interestingly, the Eigenmode method consistently calculates optima with net orbital angular momentum for circularly polarized light, for which the polarization also carries spin angular momentum. By contrast, the Eigenmode profiles calculated for linear polarizations are symmetric or anti-symmetric modes with zero net angular momentum (Fig. 3).

An analogous symmetry can be seen in Laguerre-Gaussian (LG) optics; circularly polarized beams in the LG0,1 mode have either 2*ħ* or 0 angular momentum, depending on whether the polarization and phase align or oppose each other. When the polarization and phase align for 2*ħ* angular momentum the field always has zero intensity at the focus, while for anti-aligned polarization the intensity can be peaked at the focus for sufficiently high NA^23^. We postulate that the optimized trapping profiles take advantage of similar non-paraxial effects.

**Figure 4:**
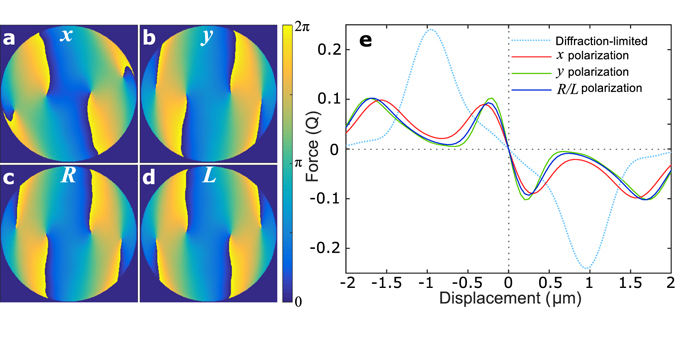
SPOT performance with different polarizations. (**a**–**d**) Example phase profiles optimized for 2 *μ*m spheres are shown for *x*, *y*, right circular (*R*) and left circular (*L*) respectively, with the trap optimized along the *x* axis. The different polarization states are optimized with slightly different profiles. Similar to the Eigenmode method, left and right circular polarizations are optimized with mirror-image profiles. The optima calculated for the different polarizations are dissimilar for all particle sizes, though the difference is more pronounced for smaller particles where the phase features are generally larger. (**e**) The resulting forces differ, with *y* polarized light achieving the strongest trap and *x* polarization the weakest, and identical forces calculated for right and left circular polarizations.

Phase optima were also calculated using SPOT for different input polarization states (Fig. 4). In contrast to the Eigenmode method, SPOT predicts modes with orbital angular momentum for all polarizations. The optimized phase profiles show slight differences for the different polarizations, and the achievable trap stiffness can vary dramatically. For 2 *μ*m particles the achievable trap stiffness with *y* polarized light is 54% higher than with *x* polarization. The phase profiles optimized for right circular and left circular polarizations are mirror images and achieve identical forces. Similar to the Eigenmode method, the handedness of the polarization state appears to establish the ideal orientation of the phase vortices.

The orientation of the vortices is slightly more important for large particles. With a 2 *μ*m particle (as in Fig. 4), right circularly polarized light achieves 15% higher stiffness than left circularly polarized light when using the phase profile optimized for right circular polarization. This polarization dependence increases to 17% and 18% with 4 and 8 *μ*m diameters respectively.

**Figure 5:**
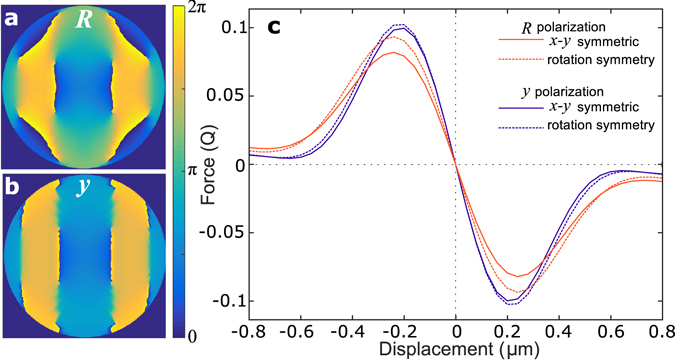
Influence of symmetry on the optimized trap. SPOT phase profiles were calculated for 2 *μ*m silica spheres while enforcing *x* − *y* symmetry for (**a**) right circular and (**b**) *y* polarizations. Note that *x* − *y* symmetry does not eliminate vortices, though it ensures that there is zero net orbital angular momentum. (**c**) Trapping forces as a function of displacement. For both polarizations the *x* − *y* symmetric profiles (solid lines) achieve poorer performance than the phase optima calculated with 180° rotational symmetry (dashed lines – profiles shown in Fig. 4b,c). This is strongest for circular polarization (light red) where *x* − *y* symmetry reduces the achievable trap stiffness by 0.86. By comparison, it only reduces stiffness by 0.99 for *y* polarization.

While the results above are rotationally symmetric, *x* − *y* mirror symmetry can also be enforced in the SPOT algorithm. This eliminates the net orbital angular momentum, though it does not remove vortices (see Fig. 5a,b). For circularly polarized light, this reduces the achievable trap stiffness by 0.86 (Fig. 5c), which further reinforces the importance of the angular momentum. This also reduces the achievable trap stiffness for linear *y* polarization, though only by 0.99.

Although optimized trapping forces have already been investigated, no work has noted the importance of vortices. Previously ref. 6 calculated phase profiles to optimize the trap, but was incapable of including vortices as the phase was represented as a spatial frequency expansion that required the phase to be continuous. Also, ref. 24 used the Eigenmode method to optimize trap stiffness, though it did not show or discuss the phase profiles.

**Figure 6:**
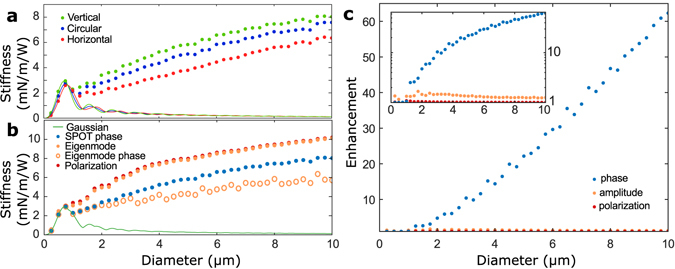
Size dependence. (**a**) Trap stiffness of diffraction-limited (solid lines) and SPOT (circles) trap as a function of particle diameter for silica spheres in water. The performance achieved with different polarizations can vary quite strongly, which is important when comparing different methods. (**b**) Trap stiffness as a function of diameter for Gaussian, SPOT, Eigenmode phase, full Eigenmode wavefront, and full polarization-amp-phase control. To allow fair comparison each data point corresponds to the polarization state that achieves highest stiffness. (**c**) The relative enhancement in trap stiffness that can be achieved using phase engineering, amplitude, and polarization. The inset shows this on a logarithmic scale. For particles larger than 1 *μ*m amplitude control offers a near-fixed enhancement with mean ± standard deviation of 1.35 ± 0.10. Polarization control offers minimal benefit, with a maximum enhancement of 1.09 at 1 *μ*m, dropping to 1.010 ± 0.003 above 5 *μ*m.

Phase-optimized SPOT traps were calculated over a range of particle sizes, and the results compared to both Gaussian trapping and the Eigenmode method (Fig. 6). The absolute trap stiffness of SPOT traps for the different polarization states across a range of particle sizes are shown in Fig. 6a, together with the trap stiffness achieved with a Gaussian trap. This shows that the *y* polarized phase-optimized trap consistently achieves the highes trap stiffness along the *x* axis. For the Gaussian trap, Mie resonances^25^ cause oscillations with opposite phase for the *x* and *y* polarizations, such that the ideal polarization swaps between the two linear polarizations periodically. Next Fig. 6b compares the trap stiffness achieved with a Gaussian, SPOT, the Eigenmode phase, the Eigenmode solution with both phase and amplitude control, as well as the Eigenmode solution with arbitrary phase, amplitude, and polarization control. To allow valid comparisons between different methods, each data point corresponds to the polarization state which achieves the highest trap stiffness for that particle. The Eigenmode solution with phase, amplitude, and polarization control is calculated in a similar manner as described above, but with the electromagnetic field represented in a mode expansion that includes arbitrary polarization.

As noted above, SPOT consistently outperforms the Eigenmode phase when applied with Gaussian amplitude. The achievable trap stiffness is highest when full phase-amplitude-polarization control is available, though this only offers a small improvement over phase-amplitude control. Likewise, phase-amplitude control offers higher stiffness than phase-only control. The relative enhancements are shown in Fig. 6c. While phase-only control can enhance stiffness by over an order of magnitude for particles larger than 3 *μ*m, addition of amplitude control or non-trivial polarizations only allow this to be further increased by a few percent. For instance, phase-control can increase stiffness for a 10 *μ*m sphere by a factor of 62, with amplitude control further increasing this by 1.24 and polarization control offering an additional 1.01 enhancement. This indicates that non-uniform polarization states such as radial or azimuthal polarizations are not important when optimizing trap stiffness; though they may play an important role for other parameters such as trap stability^26^.

**Figure 7:**
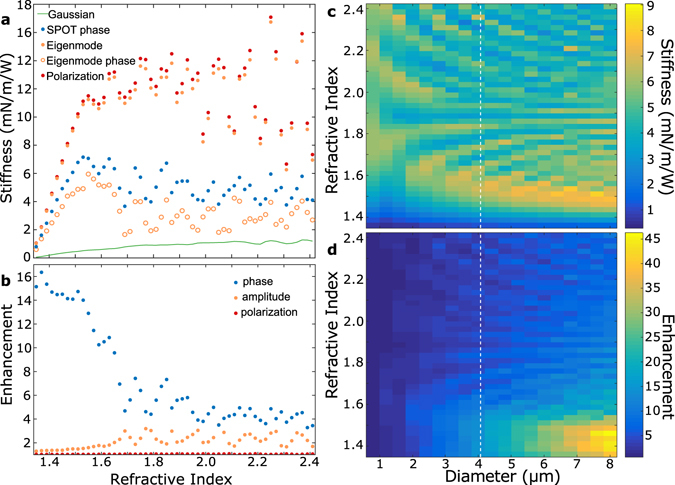
Refractive index dependence. (**a**) Trap stiffness as a function of refractive index for a 4 *μ*m microsphere, for a Gaussian trap, SPOT, Eigenmode phase, full Eigenmode wavefront, and full polarization-amp-phase control. Similar to Fig. 6b, each data point corresponds to the polarization state that achieves highest stiffness. The stiffness oscillates with changes to the Mie resonances in the particle scattering, with little clear trend above refractive index 1.55. (**b**) The relative enhancement in trap stiffness that can be achieved using phase engineering, amplitude, and polarization. The achievable enhancement from phase-engineering is largest at low refractive index where the Gaussian trap is weakest. Once again we see that polarization engineering offers minimal benefit, with 1.04 ± 0.01 increase in stiffness across the range calculated. (**c**) False-color plot of the stiffness achievable with SPOT across a range of particle diameters and refractive index, and (**d**) the relative enhancement over a Gaussian trap. The white dashed line denotes the parameters shown in (**a**,**b**).

The achievable trap stiffness was also evaluated across a range of particle refractive indices, with Fig. 7a comparing the trap stiffness achieved with the different optimization methods for a 4 *μ*m diameter sphere in water. Similar to Fig. 6b, the polarization state that provided highest trap stiffness was selected at each point. This plot shows prominent Mie resonances which strongly modulate the stiffness that can be achieved using wavefront shaping. There is little overall trend in the optimized trap stiffness as the refractive index increases beyond 1.6, but the Gaussian trap stiffness rises consistently with refractive index. As such the relative enhancements shown in Fig. 7b tends to decrease with increasing refractive index. Once again the majority of the achievable enhancement is attainable using phase-only control. Amplitude control can offer a comparable enhancement at high refractive index, while polarization control continues to offer minimal enhancement.

The trap stiffness achievable with SPOT across various particle sizes and refractive indices was calculated for *y* polarization (which is almost always optimal) and is shown in the false-color map in Fig. 7c. This map shows that Mie resonance effects appear at all particle diameters, with increasingly rapid modulations in the achievable stiffness with refractive index at larger particle diameter. This is reasonable as changes in refractive index in large particles introduce larger optical phase shifts than similar changes in small particles. While these Mie resonances introduce small regions of high trapping stiffness at high refractive index, in general it appears that there is an optimal refractive index at which the trap stiffness can be maximized in a phase-optimized trap, and that this optimal refractive index decreases with increasing particle diameter. This scaling is quite different to that of a Gaussian, such that the enhancement over a Gaussian of optimal polarization (Fig. 7d) is clearly highest for large particles and low refractive index.

It should be noted that the axial trap is not considered in the calculations here, and we have not constrained the algorithm to keep the particle at a stable axial trapping site. Some of the solutions located here are not axially stable in a high-powered single beam trap, particularly those at high refractive index. As many of the particle sizes we consider here are quite large, they can be held in an axially unstable trap by matching a repulsive optical force with the pulling force of gravity; this was the approach taken in ref. 6. Alternatively, counter-propagating fields can be used to ensure stable axial trapping.

### Applications and extensions

The enhancements discussed above could hold many important applications in the large particle regime. Note that biomolecular force sensing typically benefits from use of small particles, for which wavefront shaping offers little enhancement. However, large particles are important in applications such as fluid mechanics, levitating optomechanics, microrobotics, and some tests of fundamental particle physics. For example, hydrodynamic resonances occur when the characteristic time for the trap to center the particle is similar to the characteristic time for the perturbed fluid flow field to diffuse across the particle surface. These only become equal for a trap stiffness which is much larger than has ever been achieved in experiments, with the first observation of these resonances achieving less than 0.2 of this ref. 9. This limited the quality factor of the observed resonance to a modest 1.14, such that energy was not coherently swapped between the particle motion and the fluid flow. However, a SPOT trap of 10 *μ*m silica spheres should saturate this condition with 400 mW at the focus (see Supplementary section S2.1). This would enter a new experimental regime with optical control of microscale fluid flows, which could enable novel ways of sensing the fluid mechanics of the surrounding medium.

While so far we have exclusively optimized trap stiffness, SPOT is applicable to a large range of problems. Since the basic formalism in Eq. (1) is the same as that used in the Eigenmode method, SPOT can be applied to the same set of problems to which the Eigenmode method can be applied, which includes optimization of the magnitude of pushing or pulling forces, maximizing transmission through a scattering medium, minimizing the spot size, or maximizing the local intensity^15^. The code release^14^ includes several such examples, namely optimization of pushing, pulling and lateral force magnitude, and tracking sensitivity with a quadrant detector (based on ref. 17).

Using these, we find for instance that with a 10 *μ*m silica sphere after a 1.0NA condenser, phase optimization allows measurement sensitivity to be increased by a factor of 149. If measured at the shot noise limit, this would allow resolution of the ballistic thermal velocity using only 130 *μ*W (see Supplementary section S2.2), and with a typical 5 mW detector could allow the validity of the modified Maxwell-Boltzmann distribution in fluid to be tested with greater precision than has previously been possible^12, 27^.

Alternatively, SPOT can allow new possibilities for manipulation of high refractive index particles. Optical tweezers typically cannot stably trap a 1 *μ*m diameter sphere with refractive index above 1.8 as strong optical repulsion dominates and precludes any pulling force^18, 25^. The only reported way to overcome this is to engineer an antireflection coating on the particle to suppress the repulsive force^18^. However, phase optimization to maximize the pulling force allows pulling forces even at refractive index of 2.3 (see Supplementary section S2.3), which is the highest refractive index item to have ever been trapped using antireflection coatings. This highlights that wavefront shaping can provides access to experimental regimes that are otherwise forbidden.

## Conclusion

This paper presents a phase optimization algorithm and uses it to comprehensively study the capabilities of wavefront shaping to enhance optical trapping for the first time. We find that the phase control can increase the trap stiffness by over an order of magnitude for particle diameters over 3 *μ*m, when trapping silica spheres in water with 1064 nm light. While a diffraction limited trap achieves increasing stiffness with refractive index (neglecting the destabilizing pushing force), phase-optimized traps achieve the highest stiffness at an optimal refractive index that decreases with particle size from 1.75 for 1 *μ*m spheres to 1.45 for 10 *μ*m spheres.

The SPOT algorithm is far superior to the previous numerical approach to phase optimization used in ref. 6, both in terms of speed and performance. Previously the phase profile was constrained to be everywhere continuous which prevented the inclusion of such effects as vortices. As we have shown here, vortices can contribute to increasing the trap stiffness. As such, SPOT phase solutions can achieve up to three times higher trap stiffness. Further, this is achieved with a two order-of-magnitude reduction in the computational time. For the parameters calculated here the computational time ranged from 2–140 minutes (see Supplementary section S1.3) which compares very favorably to the 1–7 days computational time that was required for ref. 6, which until now was a major obstacle to applications of enhanced trapping and which precluded the comprehensive study of enhanced trapping. The results reported in this paper alone represent over 1000 separate optimizations, which would have been infeasible using the earlier algorithm.

The SPOT algorithm is not limited to optical forces, but can efficiently calculate phase profiles to optimize any problem that can be represented in the matrix formalism of Eq. (1). SPOT complements the Eigenmode method, which optimizes the complex wavefront in the same basic formalism. These methods are applicable to different experimental systems, with the Eigenmode method relevant to full wavefront control while SPOT assumes phase-only control, which is far more common in experiments. Together, the methods allow quantitative assessment of the relative benefits of phase, amplitude and polarization control. We envision that the SPOT algorithm will facilitate applications in optical tweezers^11^, levitating optomechanics^12, 13^, sub-wavelength imaging^28^, and light delivery through turbid media^29^.

## supplementary material

Optimizing phase to enhance optical trap stiffness: Supplementary Information
