# Supplementary material for: Optimizing phase to enhance optical trap stiffness
Source: Sci Rep. 2017 Apr 3;7:555. doi: 10.1038/s41598-017-00762-z (PMC5429609; doi:10.1038/s41598-017-00762-z)
Supplement: Supplementary file 1 — Optimizing phase to enhance optical trap stiffness: Supplementary Information [file 41598_2017_762_MOESM1_ESM.pdf]

Optimizing phase to enhance optical trap stiffness: Supplementary Information

Michael A. Taylor

S1. Implementation of the algorithm

S1.1. Code structure

The code used to implement the SPOT algorithm is available at Ref. (1). This code breaks the problem down into 7 steps:

1) Define the parameters needed for the calculation. Here the user inputs the details needed to run the optimization: the particle size and refractive index, the medium refractive index, the polarization and wavelength of the trap. If desired, one can also set the grid size and maximum order of Mie scattering for numerical evaluations; to ensure reliable results in this paper we investigated the expansion sizes that are needed for these numerical parameters (see below), and the code allows automatic selection of suitable values.

2) Calculate the Mie scattering matrix in the E(theta,phi) basis. Mie scattering is calculated in the basis of vector spherical harmonic functions using the T-matrix method implemented in the Optical Tweezers Toolbox[2]. This is then converted into the angular basis using code adapted from Ref. (3).

3) Construct the matrix that predicts optical force for any incident field. This step is described in Eq. (5) of the main text.

4) Calculate the 2D matrix that defines the spring constant. This is calculated as described in Eq. (7) of the main text.

5) Find optimized phase profile in the E(theta,phi) basis. Run the SPOT algorithm described in Eq. (3) of the main text.

6) (Optional) Find optimized wavefront using Eigenmode method. This applies the Eigenmode method to calculate the optimized wavefront for the same objective NA and polarization. This step can be skipped by setting the logical switch "compute_Eigenmode" to zero.

7) Check results with Optical Tweezers Toolbox calculations. Here the optimized fields are converted into the vector spherical harmonic basis and the trapping force and trap stiffness calculated using the Optical Tweezers Toolbox method. This is not strictly necessary but provides a separate test of the validity of the results.

S1.2. Numerical expansions

To ensure accurate results it is necessary to represent the electromagnetic field with a suitably fine angular grid, and to use a suitably large expansion of vector spherical harmonic functions for the Mie scattering. The angular grid is calculated in spherical coordinates θ, ϕ across the full spherical surface. The angular grid is evenly spaced in both θ and ϕ, with a the number of points in these axes along given by "N_theta" and "N_phi" respectively. The vector spherical harmonic functions each have two principal numbers n and m, with the azimuthal parameter m ranging from $-n$ to n. This expansion is truncated at "Nmax" which is the highest n included in the analysis.

To check the requirements of these, we first calculated optimized phase profiles using a range of Nmax. The trap stiffness achieved with each optimized phase profile was then tested. To avoid truncation errors when testing the profiles, optical forces were always evaluated using a larger numerical Nmax and angular grid than used in the SPOT optimization. This shows that the trap stiffness achieved with SPOT depends strongly on Nmax when Nmax is low, but once Nmax is suitably large there is no further benefit to increasing Nmax (see Fig. S1). The dependence of SPOT stiffness on Nmax was evaluated for a range of particle sizes, and an approximate rule was composed which would always keep Nmax larger than the minimum required value. This rule appears reasonable when considering the scattering amplitudes of the T-matrix; the rule applied here only truncates vector spherical harmonic terms for which the scattered amplitude is below 10^{-5} of the incident amplitude.

Next we calculated SPOT phase profiles using a range of N_theta and N_phi and tested the trap stiffness achieved, again using a larger numerical Nmax and angular grid for force calculations to avoid truncation errors. Once more this revealed that it was crucial to use a suitably large angular mesh, but that once this was reached there was little benefit to further increasing the mesh size. The required mesh size was tested for a range of particle sizes, and rules were composed which would always keep N_theta and N_phi well above the minimum requirements (Fig. S1). These three rules are included in the attached code so the numerical parameters can be chosen automatically. Note that the particle refractive index is not explicitly considered in these rules; it was found that moderate variations in refractive index had no effect on the required numerical expansions. If trapping very high refractive index particles ($n > 2$), however, a larger numerical expansion may be needed.

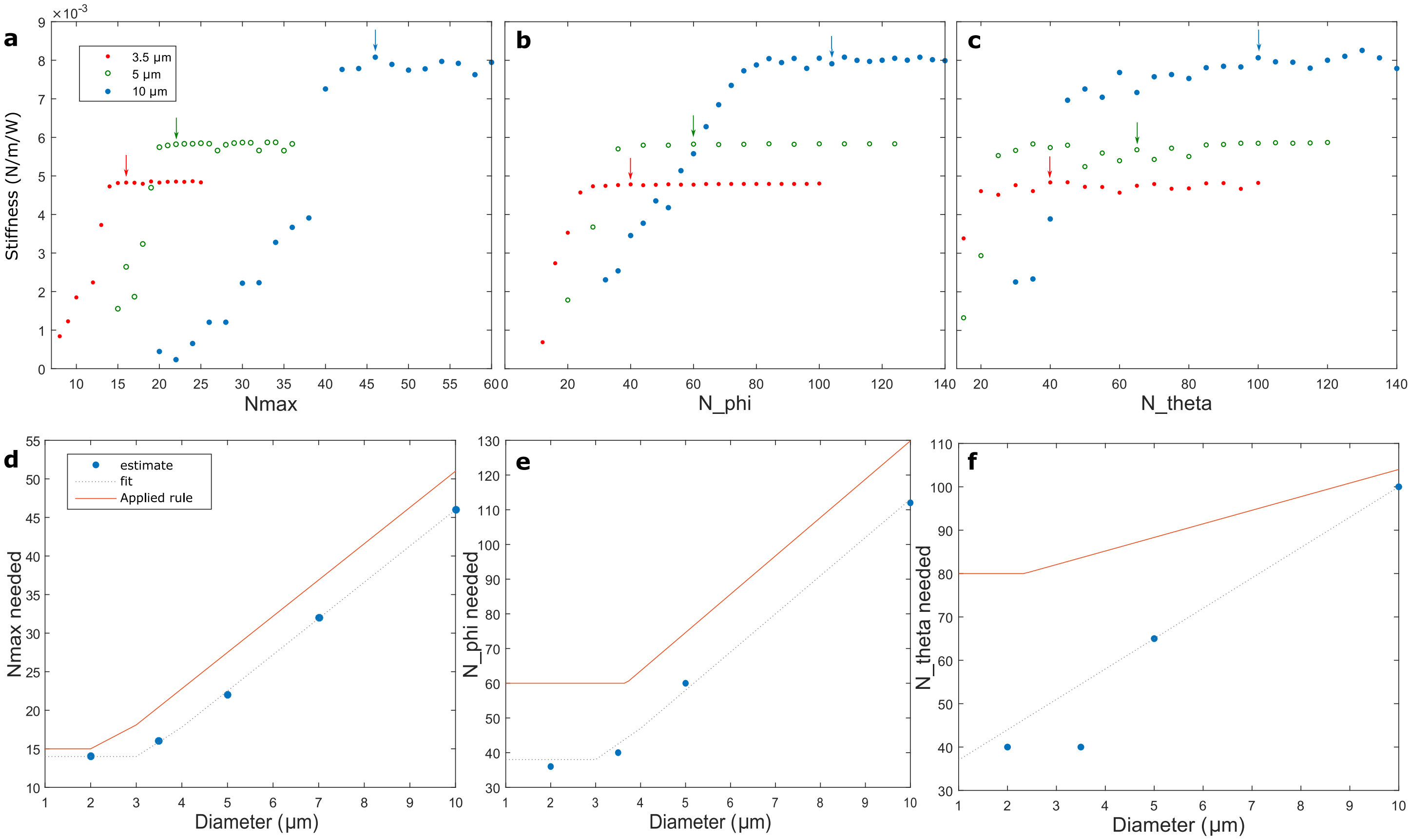

Fig. S1: **Numerical expansion sizes needed.** **a,** The spring constant achieved with SPOT optimization using a range of values of Nmax, for 3.5 μm, 5 μm and 10 μm diameter particles. If Nmax is too low SPOT converges to a poor profile, but above some threshold (approximately indicated with arrows) no further benefit is found to increasing Nmax. Similar tests are shown in **b** and **c** for different angular grids N_phi and N_theta respectively. **d,** The estimated minimum required Nmax as a function of particle diameter. This was tested at five separate particle diameters, comprising the three tests shown in **a** as well as similar tests at 2 μm and 7 μm diameter. Based on these estimates, a rule was composed (red line) to automatically select an Nmax value that always remains safely over the minimum requirements. Similar analyses are shown in **e** and **f** for different angular grids N_phi and N_theta respectively. These rules allow automatic selection of suitable numerical expansions based on particle diameter which should always remain safely over the minimum required values.

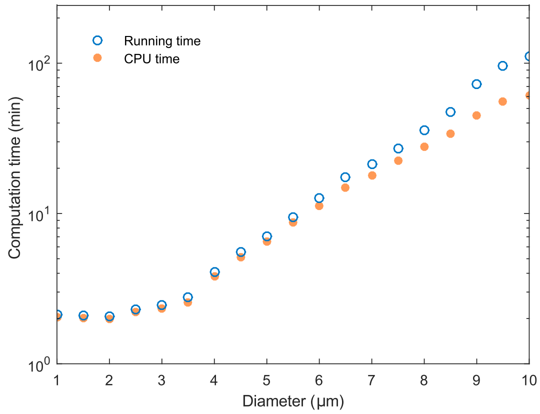

Fig. S2: **Time to run code.** The time taken to complete the SPOT algorithm as a function of particle diameter, using silica microspheres (n=1.46) in water (n=1.33), trapped using 1064 nm light with circular polarization, and calculated on the laptop computer described in the text of section S1.3. The results here show both the actual time taken to complete the code (open), and the "CPU time" (filled), which is the amount of time that the code occupies the computer CPU.

S1.3. Computational requirements

One of the key advantages of the SPOT algorithm is its computational efficiency, especially when compared with the steepest descent approach used previously in Ref. (4). The paper reports optimization results across a range of different refractive indices with particle diameters ranging from 1–10 μm. Most of these results were calculated using a laptop computer with 8GB of RAM and a further 18GB of virtual memory available, 2.2GHz dual core CPU, and running Matlab R2015a. The running time of the code was tested using this laptop computer and for silica particles of diameter 1–10 μm. The Eigenmode algorithm was not included in the running time, with the logical switch "compute_Eigenmode" set to zero to skip calculation of Eigenmodes (see section S1.1). The resulting computation time is shown in Fig. S2 both in terms of the actual time taken to complete the code and the amount of time that the code occupies the computer CPU. Since the computer has dual processors, the CPU time reported by Matlab has been halved to allow easy comparison with the actual running time. At small particle sizes there is little difference between the actual and CPU times, indicating that the code fully occupies the CPU cores for almost all of the running time. At larger particle diameter, however, the running time increases much more than the CPU time, indicating that memory becomes an increasingly limiting factor to the speed of the code.

The memory used when running the SPOT code scales approximately with the fourth power of particle diameter. The code works in two spatial mode bases; the vector spherical harmonic functions, and the plane-wave basis. In both cases, the number of discrete spatial modes included in the computation scales as the particle size squared. As noted in section S1.2, for large particles the required Nmax, N_theta, and N_phi all increase linearly with particle size. The plane wave basis size is simply N_theta $\times$ N_phi, which is proportional to the particle size squared; while the total number of vector spherical harmonic functions is given by 2 Nmax^2 + 4 Nmax, which also grows as particle size squared. The SPOT algorithm relies on square matrices where the number of columns is the number of spatial modes (see Eqs. (1), (5) of the main text), so the arrays used in the computation have size which scales as particle size to the fourth power. The computation is generally much more efficient with small arrays that are held within the computer RAM, though larger arrays can also be manipulated at lower speed using the hard drive for virtual memory. The virtual memory was found to be very important for large diameter calculations. If less virtual memory is available the code runs more slowly, and if the virtual memory is insufficient the code fails to run.

Although the laptop computer is capable of performing all calculations reported in this paper, the code was found to run much faster on more powerful computers, particularly for large particle size. To save time, many of the results with particle diameter larger than 7 μm were calculated on more powerful computers.

S2. Proposed Applications

The main text briefly discusses two specific applications. Here some additional details are given on the performance criteria of those applications, and how the optimized trap can reach these criteria.

S2.1. Hydrodynamic resonance

Hydrodynamic resonances occur when the characteristic time for the trap to center the particle is similar to the characteristic time for the perturbed fluid flow field to diffuse across the particle surface[5]. The trapping timescale is given by $\tau_k = \gamma/\kappa$, where κ the spring constant, $\gamma = 6\pi\eta R$ is the Stokes friction coefficient, η is the fluid viscosity and R the particle radius. The inverse of this timescale is the *corner frequency*, which is more familiar to the optical tweezers community. The timescale of fluid flow is given by $\tau_f = R^2\rho_f/\eta$, with ρ_f the fluid density[5]. These two timescales equal for an optimal trap stiffness of

$$\kappa_{\text{opt}} = \frac{6\pi\eta^2}{R\rho_f}. \tag{1}$$

As this scales inversely with particle size, hydrodynamic resonances can be induced at lower trap stiffness using larger spheres. The main text discusses use of 10 μm diameter spheres (5 μm radius) in water. At room temperature (298K) $\eta = 8.9 \times 10^{-4}$, which leads to an optimal trap stiffness of 3.0 mN/m. The SPOT trap for 10 μm silica microspheres achieved 8.0 mN/m/W, such that this limit is reached with 380 mW. However, the precise value of this could vary wildly with experimental parameters. For instance, the viscosity of water varies with temperature, which causes the required power to change by 26% for a 5K temperature difference. As such, the required power is better specified as 400 mW with one significant figure.

S2.2. Brownian velocity

Another discussed application is to characterize the instantaneous thermal velocity of microspheres in water. The extreme measurement sensitivity required to measure instantaneous velocity in liquid was only achieved in 2014[6]. So far the experimental data agrees with the modified Maxwell-Boltzmann equation, where the effective particle mass is includes both the particle mass and the mass of a co-moving fluid envelope, $m^* = 4/3\pi R^3\rho_p + 2/3\pi R^3\rho_f$. Experimental data so far agrees with the modified Maxwell-Boltzmann equation in acetone, within the limits of the achievable signal-to-noise ratio[6,7]. If a violation exists, it can only be observed with improved measurement precision. Further, no experimental data to date has achieved the precision required to quantitatively test the modified Maxwell-Boltzmann equation in water. Acetone has much lower viscosity than water, which eases the sensitivity required to resolve instantaneous thermal velocity.

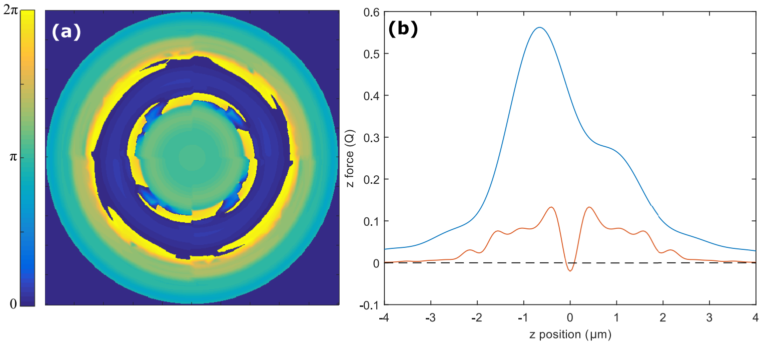

Fig. S3: **SPOT optimization of pulling force with high refractive index (2.3) particle. a,** The phase profile optimized for negative force magnitude at $z = 0$. **b,** The resulting axial force as a function of position. The blue curve shows the force profile with a Gaussian trap, which is everywhere strongly repulsive; and the orange curve shows the SPOT force profile, which allows a weak pulling force over a small range near the focus.

Here we use the criterion to resolve instantaneous Brownian velocity which was earlier introduced in Ref. (8). Briefly, any particle momentum dissipates over a characteristic timescale of $\tau_p = m^*/\gamma$. To resolve the Brownian velocity the motion should be measured with a minimum time resolution of $\tau_p/10$. On average the particle will move by $v_{rms}\tau_p/10$ between each time point, and if we aim to measure this with 10% position uncertainty we must achieve position resolution of $\Delta x \leq v_{rms}\tau_p/10$, with $v_{rms} = 2kT/m^*$ the root-mean-square velocity, k Boltzmann's constant and T the temperature. Together, this specifies both a minimum bandwidth and a minimum sensitivity in m $\text{Hz}^{-1/2}$ to resolve Brownian velocity. Previously, Refs. (6) and (7) respectively exceeded the required sensitivity by a factor of 10 and 30 with Barium Titanate spheres in acetone. While both papers also presented experiments in water, neither reached the minimum criteria described here. The data recorded in water did not show agreement with the predictions of the modified Maxwell-Boltzmann equation, though this may simply result from inadequate measurement precision[7].

The dependence on effective mass allows coarser sensitivity as the particle size increases. The detector sensitivity needed to resolve Brownian velocity in water at 298K with a 10 μm silica sphere is 13 fm $\text{Hz}^{-1/2}$, while a 1 μm sphere requires a far superior 0.38 fm $\text{Hz}^{-1/2}$.

To optimize a phase profile for measurement sensitivity, we adapt the detection algorithm introduced in Ref. (3). All intensity outside of the condenser angular is neglected, and the light within the aperture is directed onto a quadrant detector. The resulting signal s along x is given by the total power with positive lateral momentum ($p_x > 0$) minus the total power with negative lateral momentum ($p_x < 0$). To optimize the detection sensitivity, the change in signal with particle displacement $G_x = \partial s/\partial x$ should be maximized. The shot-noise limit to sensitivity can then be directly estimated[3] from G_x. Implementing the SPOT algorithm for detection sensitivity with 1.0 condenser NA, we find that phase optimization can increase G_x by a factor of 149, which provides a corresponding increase in sensitivity. If this is achieved with shot-noise limited detection the criteria above is saturated with 130 μW of detected light.

S2.3. High-index pulling force

As noted in the main text, the force magnitude can be optimized by using the matrix A from Eq. (5) as the matrix M in Eq. (3). We used this to optimize the magnitude of the force along the axial z axis, specifically maximizing the negative pulling force. This was applied to a 1 $/mu$m diameter sphere with refractive index 2.3, corresponding to anatase Titania. The optimized profile and the resulting forces are shown in Fig. S3, which shows that phase optimization allows a pulling force. This example demonstrates pulling forces at a refractive index where such forces are usually precluded. As the particle is pulled along the z axis this transitions to a pushing force, so this profile achieves an axial trap. However, the lateral forces tend to repel the particle from the focus, so this profile does not achieve a stable trap.

References

[1] Taylor, M. A. Sequential phase optimization technique. https://github.com/michael-a-taylor/SPOT (2016).

[2] Nieminen, T. *et al.* Optical tweezers computational toolbox. *J. Opt. A: Pure Appl. Opt.* **9**, S196 (2007).

[3] Taylor, M. A. & Bowen, W. P. A computational tool to characterize particle tracking measurements in optical tweezers. *J. Opt.* **15**, 085701 (2013).

[4] Taylor, M. A., Waleed, M., Stilgoe, A. B., Rubinsztein-Dunlop, H. & Bowen, W. P. Enhanced optical trapping via structured scattering, *Nat. Photon.* **9**, 669–673 (2015).

[5] Franosch, T. *et al.* Resonances arising from hydrodynamic memory in Brownian motion. *Nature* **478**, 85–88 (2011).

[6] Kheifets, S., Simha, A., Melin, K., Li, T. & Raizen, M. G. Observation of Brownian motion in liquids at short times: instantaneous velocity and memory loss. *Science* **343**, 1493–1496 (2014).

[7] Mo, J., Simha, A., Kheifets, S. & Raizen, M. G. Testing the Maxwell-Boltzmann distribution using Brownian particles. *Opt. Express* **23**, 1888–1893 (2015).

[8] T. Li. "Fundamental tests of physics with optically trapped microspheres," (Springer, 2013).
